# Evidence for a Novel Care Management Program for Low-Income Older Adults

**DOI:** 10.1093/geroni/igaa057.2917

**Published:** 2020-12-16

**Authors:** Rachel Lessem, Margaret Danilovich

**Affiliations:** 1 CJE SeniorLife, Chicago, Illinois, United States; 2 CJE SeniorLife, Evanston, Illinois, United States

## Abstract

The purpose of this study was to evaluate the implementation and effectiveness of a novel care management program for low income older adults in Chicago. Older adults (n=200) who had annual income below $31,225 but about the state level for home and community based services were received care management. Program participants completed a battery of assessments (UCLA Loneliness Scale, single item Quality of Life and Physical Health scales, and Nutritional assessment) at initial assessment and 1-year follow-up. We also conducted interviews with clients and care managers. We used a t-test to evaluate participant outcomes and coded qualitative data to identify themes. Results showed no significant differences between baseline and 1 year follow-up indicating that this care management program kept participants stable. Only 5 of 200 (2.5%) of clients transitioned to a nursing home. This study contributes important results on a novel program to sustain vulnerable older adults in the community.

